# Modified Gerchberg–Saxton (G-S) Algorithm and Its Application

**DOI:** 10.3390/e22121354

**Published:** 2020-11-30

**Authors:** Tieyu Zhao, Yingying Chi

**Affiliations:** Information Science Teaching and Research Section, Northeastern University at Qinhuangdao, Qinhuangdao 066004, China; chiyingying@neuq.edu.cn

**Keywords:** G-S algorithm, single-phase retrieval algorithm, double-phase retrieval algorithm, multiple-phase retrieval algorithm, image encryption

## Abstract

The Gerchberg–Saxton (G-S) algorithm is a phase retrieval algorithm that is widely used in beam shaping and optical information processing. However, the G-S algorithm has difficulty obtaining the exact solution after iterating, and an approximate solution is often obtained. In this paper, we propose a series of modified G-S algorithms based on the Fresnel transform domain, including the single-phase retrieval (SPR) algorithm, the double-phase retrieval (DPR) algorithm, and the multiple-phase retrieval (MPR) algorithm. The analysis results show that the convergence of the SPR algorithm is better than that of the G-S algorithm, but the exact solution is not obtained. The DPR and MPR algorithms have good convergence and can obtain exact solutions; that is, the information is recovered losslessly. We discuss the security advantages and verification reliability of the proposed algorithms in image encryption. A multiple-image encryption scheme is proposed, in which *n* plaintexts can be recovered from *n* ciphertexts, which greatly improves the efficiency of the system. Finally, the proposed algorithms are compared with the current phase retrieval algorithms, and future applications are discussed. We hope that our research can provide new ideas for the application of the G-S algorithm.

## 1. Introduction

Phase retrieval was first proposed to solve the imaging problem of an electron microscope. Gerchberg and Saxton addressed the issue of the wavefront phase of the light field via numerical iteration using the intensity of the image plane and the diffraction plane [1,2]. Since the successful solution of this problem, phase retrieval has aroused significant research interest and has been extended to many engineering fields, such as X-ray imaging, astronomical imaging, adaptive optics, and binary optical design [3,4,5,6,7,8,9]. Gerchberg and Saxton first proposed a numerical algorithm in 1972, known as the G-S algorithm [2]. The G-S algorithm has groundbreaking significance in addition to a number of shortcomings. Some of these shortcomings are inherent to the solution of inverse problems. For example, the Fourier transform amplitude of a function to a function is a many-to-one mapping, and finding a function from the Fourier transform amplitude will inevitably have multiple solutions. The other issue arises from the algorithm itself. For example, after the first few iterations, the convergence speed of the G-S algorithm slows down or even stagnates. Two important improvements to the G-S algorithm were made by Misell and Fienup. In 1973, Misell proposed that iterating between two images with different levels of defocus can improve the accuracy and convergence of the algorithm [10]. Misell’s algorithm is more practical, and provides a reference for the improvement of the G-S algorithm. In 1982, Fienup noted that G-S is an error descent algorithm, and its essence is the same as the fastest descent algorithm [11]. To solve the problem of stagnation of the G-S algorithm, Fienup proposed a hybrid input-output (HIO) algorithm based on nonlinear control. The HIO algorithm effectively improves the convergence effect and is widely used.

Due to the correlation of image pixels, the G-S algorithm can obtain a clear target image, and is thus widely used in image encryption. In 1996, Johnson et al. proposed a new optical image encryption scheme, which encrypts a plaintext image into two phases [12]. In the same year, Wang et al. used the G-S algorithm to encrypt a plaintext image into the phase of the Fourier spectrum [13]. In addition, Zalevsky et al. extended the G-S algorithm to the fractional Fourier transform domain, which further promoted the application of the algorithm [14]. Image encryption schemes based on the phase retrieval algorithm are generally implemented with the help of a photoelectric hybrid system so that both the optical system and the computer can be used in the recovery process of the plaintext [15,16,17,18,19,20,21]. Some multiple-image encryption schemes have been proposed to greatly improve the efficiency of the system [22,23]. Moreover, the G-S algorithm has been expanded to three-dimensional space with greater flexibility, which can achieve image encryption and identity verification to ensure security [24,25,26]. These developments mainly involve the application of the G-S algorithm in symmetric cryptosystems; however, it is also widely used in key distribution [27] and asymmetric cryptosystems [28,29,30,31]. In an asymmetric cryptosystem, the encryption key and the decryption key are independent of each other, which is more satisfactory for practical applications. In 2015, Zhao et al. proposed an asymmetric image encryption system using human biometrics combined with the G-S algorithm. A fingerprint can be used to verify the authenticity of the ciphertext in this system [32]. In addition, the G-S algorithm can be well combined with other encryption technologies to improve security [33,34]. Image encryption technology is time effective. To ensure the security of information, recently, some generalized G-S algorithms have been applied to image encryption [35,36,37,38].

The application of the G-S algorithm in image encryption has greatly promoted its theoretical development [39]. The obtained phase is an approximate solution due to the attributes of G-S, that is, lossless plaintext cannot be recovered. In this paper, we attempt to modify the G-S algorithm and apply it to image encryption. The research results are as follows:

(a) A single-phase retrieval algorithm based on the Fresnel transform domain is proposed and compared with the G-S algorithm.

(b) We further propose a double-phase retrieval algorithm and a multiple-phase retrieval algorithm, and prove that they have a good convergence effect.

(c) A multiple-image encryption scheme is proposed to greatly improve the efficiency of the system.

The remainder of this paper is organized as follows. The preliminary knowledge is described in Section 2. Section 3 proposes the modified G-S algorithm. The convergence is discussed in Section 4. Section 5 analyzes the security and reliability of the proposed algorithm, and further presents multiple-image encryption. The proposed algorithms are compared with the current phase retrieval algorithms, and the future applications are discussed in Section 6. Finally, the conclusions are presented in Section 7.

## 2. Preliminaries

### 2.1. G-S Algorithm

**Figure 1:**
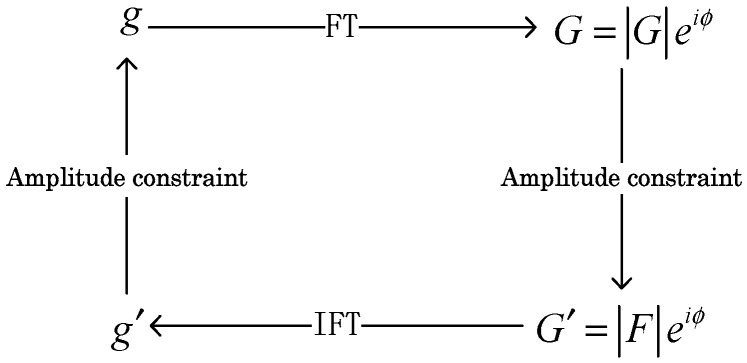
Gerchberg–Saxton (G-S) algorithm. FT—Fourier transform, and IFT—inverse Fourier transform.

In 1972, Gerchberg and Saxton first proposed an algorithm for solving the phase recovery problem in the study of electron microscope imaging [2]. Here, we briefly introduce the process of the G-S algorithm, as shown in Figure 1. First, an initial guess $g_{0}\left(x,y\right)$ is input, and the amplitude |G(u,v)| is obtained by the Fourier transform. Next, the amplitude |G(u,v)| is replaced by a known function |F(u,v)| to form a new function. Then, the estimated square function $g^{\prime }\left(x,y\right)$ is obtained by the inverse Fourier transform, and the input value g(x,y) of a new iteration is formed with known constraints.

In the given initial phase distribution, the amplitude distributions of the input and the output are successively iterated, which can determine the phase distribution of the input. The G-S algorithm can be implemented using Equations (1)–(4).

(1) $$G_{k-1}\left(u,v\right)=FT\left[g_{k-1}\left(x,y\right)\right]=\left|G_{k-1}\left(u,v\right)\right|e^{i\phi_{k-1}\left(u,v\right)},$$

(2) $$G_{k}^{\prime }\left(u,v\right)=\left|F\left(u,v\right)\right|e^{i\phi_{k-1}\left(u,v\right)},$$

(3) $$g_{k}^{\prime }\left(x,y\right)=IFT\left[G_{k}^{\prime }\left(u,v\right)\right]=\left|g_{k}^{\prime }\left(x,y\right)\right|e^{i\phi_{k}\left(x,y\right)},$$

(4) $$g_{k}\left(x,y\right)=\left|g\left(x,y\right)\right|e^{i\phi_{k}\left(x,y\right)}.$$

k=1,2,⋯,n

The iterations of the algorithm can be controlled using the mean squared error (MSE) or the correlation coefficient (CC). The main principle is to control the iterative process by calculating the difference between the amplitude of the spectrum surface and the preset amplitude. The iteration stops when the MSE or CC satisfies a certain preset condition; otherwise, the next iteration starts.

**Figure 2:**
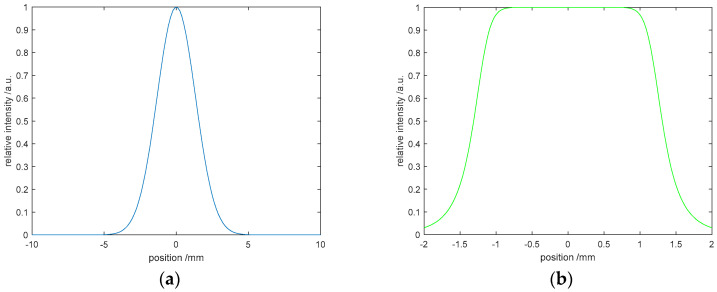

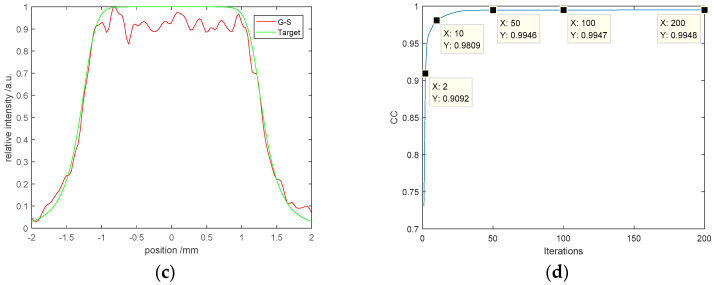
Numerical simulation of the G-S algorithm: (**a**) Gauss function curve, (**b**) target function curve, (**c**) comparative analysis, and (**d**) correlation coefficient curve.

Next, we conducted a comparative analysis with a two-dimensional function graph. The function graph of the Gaussian beam is shown in Figure 2a, and Figure 2b is the function graph of the target light intensity. In the simulation experiment, the G-S algorithm performed 200 iterations in total, and the comparison results are shown in Figure 2c. The red curve is obtained again, and the green curve is the target function curve. Figure 2d is the CC curve. The closer the CC is to 1, the better the convergence effect of the algorithm. Figure 2d shows that the initial convergence speed is relatively fast. After 50 iterations, the convergence speed is very slow, and the phenomenon of stagnation occurs.

As in the above analysis, when the G-S algorithm is applied to beam shaping, the problem arises that the convergence stagnates.

### 2.2. Application of G-S Algorithm in Image Encryption

The G-S algorithm is widely used in image encryption. Due to the correlation between the pixels of an image, a plaintext image can be recovered well. Here, we introduce the most common and simplest encryption scheme. In the encryption process, the optical attributes involved usually include the wavelength, focal length, diffraction distance, phase, etc., which can be used as the keys.

**Figure 3:**
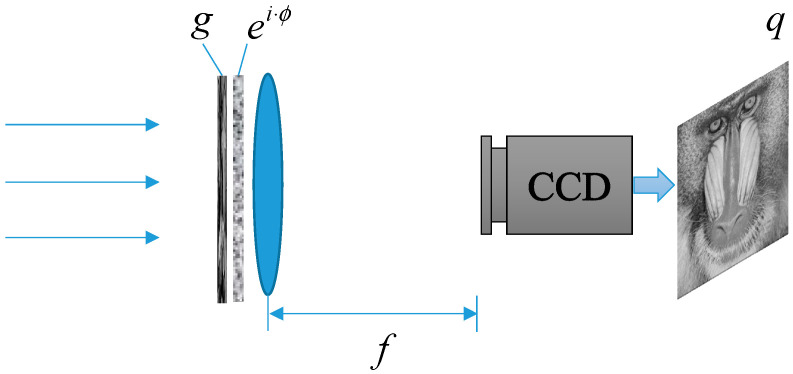
The G-S algorithm applied to image encryption. f: the focal length. CCD: charge coupled device.

The principle of image encryption based on the G-S algorithm is shown in Figure 3. Here, g is a known amplitude image, assuming there is phase $e^{i\cdot \phi }$ (ciphertext). Then, the light intensity detector at the receiving end can obtain the image q (plaintext).

**Figure 4:**
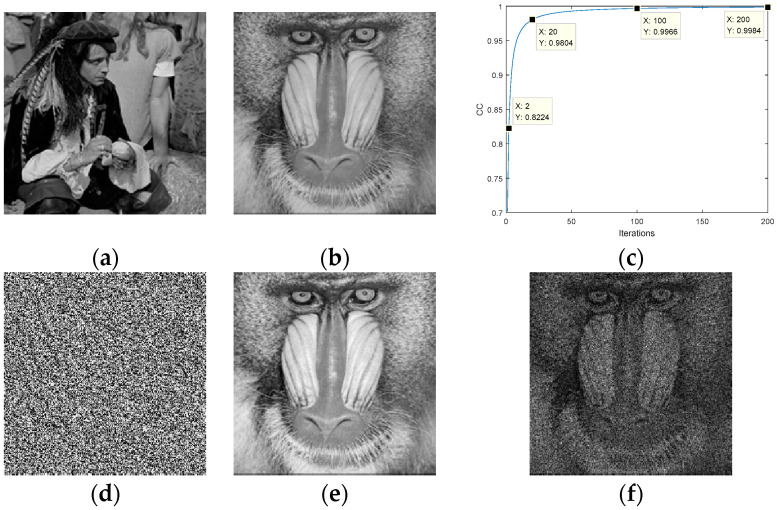
G-S algorithm applied to image encryption: (**a**) input amplitude image, (**b**) target amplitude image, (**c**) number of iterations, (**d**) ciphertext, (**e**) the recovered plaintext image, (**f**) the recovered plaintext image without amplitude.

Next, we used the G-S algorithm to obtain the phase $e^{i\cdot \phi }$ (ciphertext). In the encryption process, we used the G-S algorithm for 200 iterations, and the numerical simulation results are shown in Figure 4. Figure 4a,b are a known input amplitude image and a target amplitude image, respectively. The CC was selected as the criterion for judging the convergence of the iterations. The value of CC was 0.9984 after 200 iterations. The transformation curve of CC is shown in Figure 4c.

The phase $e^{i\cdot \phi }$ obtained is shown in Figure 4d, which is also ciphertext. The decryption process can be completed either by an optical system or by a computer. The phase $e^{i\cdot \phi }$ was placed in the optical system of Figure 3, and the plaintext can be obtained directly at the receiver. In the computer, the receiver can obtain the plaintext image via the operation $\left|FT\left(g\cdot e^{i\phi }\right)\right|$. The recovered plaintext image is shown in Figure 4e.

It is well-known that the G-S algorithm has difficulty obtaining an exact solution, but the recovered image is no different from the original image visually. We attempted to recover the plaintext using the operation $\left|FT\left(e^{i\phi }\right)\right|$ (only phase), and the result is shown in Figure 4f. The result shows that we can still recognize the plaintext image information, which indicates that the phase is dominant in image reconstruction.

### 2.3. The Importance of the Phase

**Figure 5:**
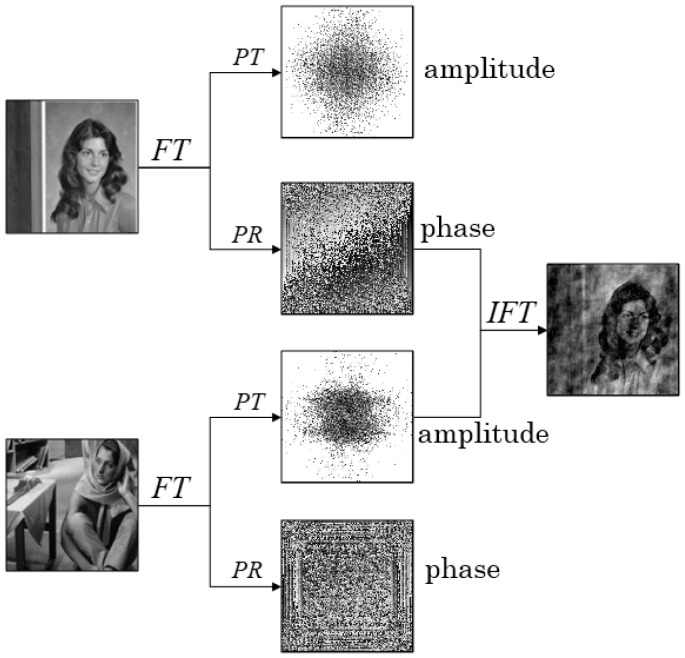
The importance of the phase. PT denotes the phase truncation operation, PR—the phase reservation observation, FT—Fourier transform, and IFT—inverse Fourier transform.

The phase is very important in signal processing due to its ability to reconstruct a signal completely [40,41]. Similarly, the phase is also very important in image processing, as shown in Figure 5. The phase and the amplitude were extracted by applying the Fourier transform to the images. We combined the phase of one image with the amplitude of another. Then, the new image was obtained using the inverse Fourier transform, and we can recognize the image information. This shows the importance of the phase in image processing. Here, *PT* and *PR* denote the phase truncation and phase reservation operations, respectively. For any complex amplitude $F=\left|F\right|e^{i\cdot \phi }$,

(5) PT(F)=|F|,

(6) $$PR\left(F\right)=e^{i\cdot \phi }.$$

This analysis shows that the phase is dominant in image reconstruction. Inspired by this, we attempted to use the phase for image reconstruction, and then propose the modified G-S algorithm.

## 3. Modified G-S Algorithm

In this section, we propose three modified G-S algorithms in a progressive relationship. They are the single-phase retrieval (SPR) algorithm, the double-phase retrieval (DPR) algorithm, and the multiple-phase retrieval (MPR) algorithm.

### 3.1. Single-Phase Retrieval Algorithm

**Figure 6:**
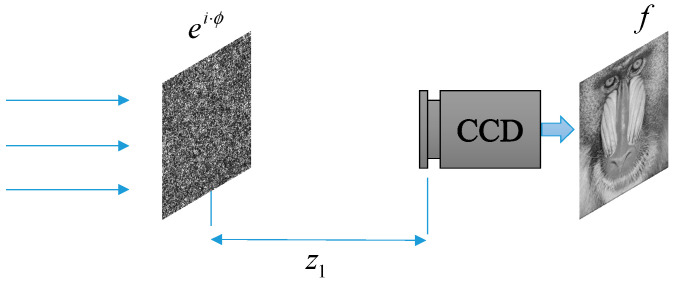
The optical system of single-phase modulation: $z_{1}$ is the diffraction distance; CCD—charge coupled device.

Our idea is shown in Figure 6. The modified G-S algorithm is applied to the Fresnel transform domain, which is more flexible; and the diffraction distance can be used as a key to improve security. We try to recover the target image using only a single phase during the iteration.

Obtaining the phase $e^{i\cdot \phi }$ is the core problem of our research. Therefore, we refer to the idea of the G-S algorithm to propose an SPR algorithm based on the Fresnel transform domain.

For the initial random phase mask $e^{i\cdot \phi_{0}}$ with $\phi_{0}\in \left(-\pi ,\pi \right)$, the complex amplitude $S^{0}=\left|S^{0}\right|\cdot e^{i\cdot \arg\left(S^{0}\right)}$ is obtained after the Fresnel transform. Then, the amplitude $\left|S^{0}\right|$ is replaced to obtain a new complex amplitude $S^{1}=f\cdot e^{i\cdot \arg\left(S^{0}\right)}$. Next, the function $F^{1}=\left|F^{1}\right|\cdot e^{i\cdot \arg\left(F^{1}\right)}$ is obtained after the inverse Fresnel transform. The extracted phase $e^{i\cdot \arg\left(F^{1}\right)}$ replaces the initial phase $e^{i\cdot \phi_{0}}$ and enters the next iteration. The iterations continue until the iterative result meets the preset requirements. The SPR algorithm is shown in Equations (7)–(10):

(7) $$S^{k-1}=FrT_{z_{1}}\left(e^{i\cdot \phi^{k-1}}\right)=\left|S^{k-1}\right|\cdot e^{i\cdot \arg\left(S^{k-1}\right)},$$

(8) $$S^{k}=f\cdot e^{i\cdot \arg\left(S^{k-1}\right)},$$

(9) $$F^{k}=FrT_{-z_{1}}\left(S^{k}\right)=\left|F^{k}\right|\cdot e^{i\cdot \arg\left(F^{k}\right)},$$

(10) $$e^{i\phi^{k}}=e^{i\cdot \arg\left(F^{k}\right)},$$

k=1,2,⋯,n

**Figure 7:**
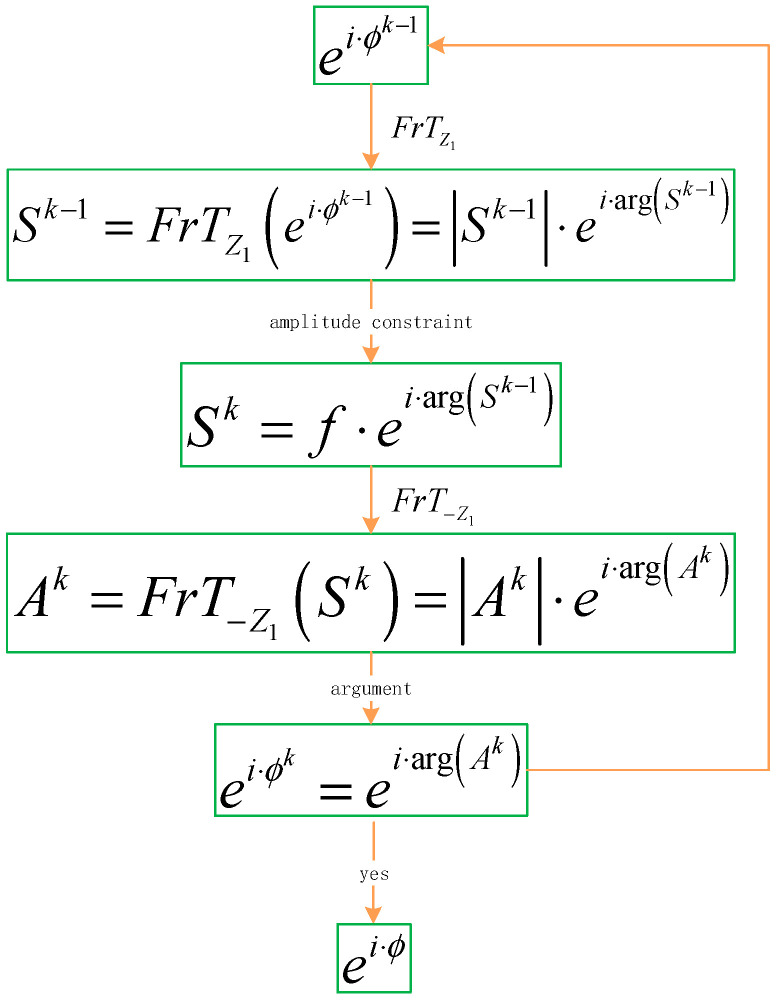
The flowchart of the single-phase retrieval (SPR) algorithm.

Here, $FrT_{z_{1}}\left(\cdot \right)$ denotes the Fresnel transform with distance *z*_1_, f denotes a known image, |·| is the modulus operation, and arg(·) is the argument function. It is known that image f is transformed into a phase encoding $e^{i\cdot \phi }$ through the above iterative process. When the phase encoding $e^{i\cdot \phi }$ passes through the optical system, the image can be directly captured by the light intensity detector, as shown in Figure 6. In addition, the image can also be obtained by the operation $f=|FrT_{z_{1}}(e^{i\phi })|$. The flowchart of the SPR algorithm is shown in Figure 7.

We use the CC as a criterion for judging the iterative convergence. f(x,y) denotes the known image, and $\hat{f}(x,y)$ denotes the recovered image. Then, the CC can be expressed as follows:

(11) $$CC=\frac{COV[f(x,y),\hat{f}(x,y)]}{\sigma_{f}\cdot \sigma_{\hat{f}}}.$$

Here, COV is the covariance, and σ is the variance. The closer the value of the correlation coefficient to 1, the better the convergence.

**Figure 8:**
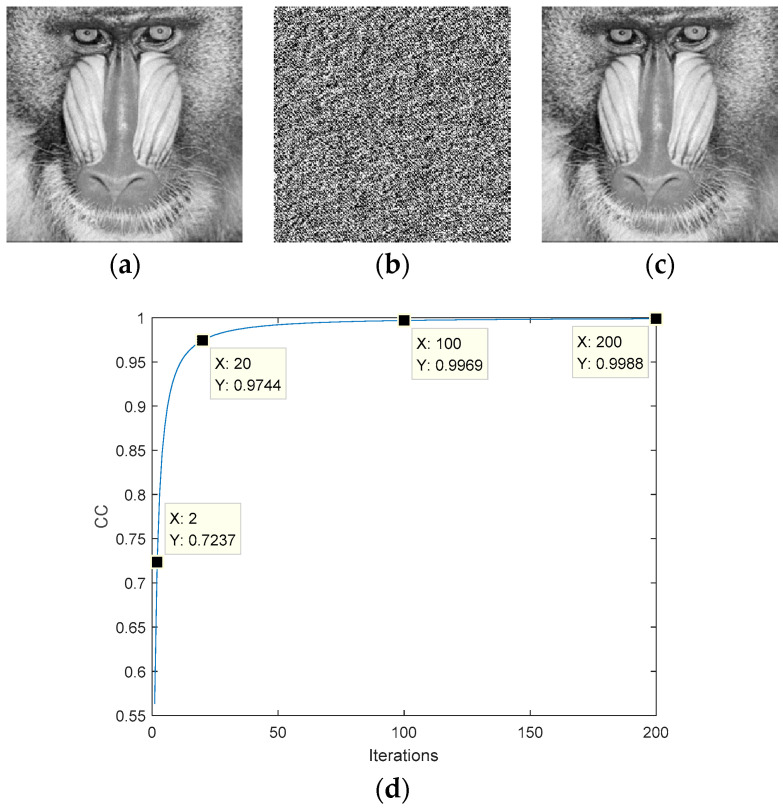
Simulation of the single-phase retrieval algorithm: (**a**) target amplitude image, (**b**) ciphertext, (**c**) the recovered plaintext image, (**d**) the transformation curve of the correlation coefficient (CC).

The simulation results are shown in Figure 8. Figure 8a is the target image. The phase obtained after the iterations is shown in Figure 8b. Figure 8c is the recovered image, and the reconstruction process can be completed using the optical system of Figure 6 or the operation $\left|FrT_{z_{1}}\left(e^{i\phi }\right)\right|$. Similar to the G-S algorithm, the SPR algorithm converges quickly in the early stages of the iterative process, but is slow in the later stages. The CC after 200 iterations is 0.9988.

### 3.2. Double-Phase Retrieval Algorithm

**Figure 9:**
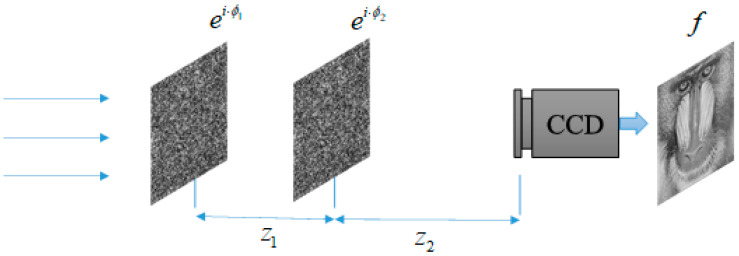
The optical system of double-phase modulation.

Here, we try to iterate using two phases, and our idea is shown in Figure 9. When the two phase encodings are arranged in order, the light intensity detector at the output can directly acquire the recovered image.

Based on the research in Section 3.1, we present the DPR algorithm. The mathematical expression of its detailed process is as follows.

The initial random phases are $e^{i\cdot \phi_{1}^{0}}$ and $e^{i\cdot \phi_{2}^{0}}$, where $\phi_{1}^{0},\phi_{2}^{0}\in \left(-\pi ,\pi \right)$ are the random matrices. Then, the wavefront function of the CCD can be expressed as:

(12) $$S^{0}=FrT_{z_{2}}\left[FrT_{z_{1}}\left(e^{i\cdot \phi_{1}^{0}}\right)\cdot e^{i\cdot \phi_{2}^{0}}\right].$$

Assuming that there is a phase $e^{i\cdot \phi_{2}^{1}}$, it satisfies Equation (13):

(13) $$f\cdot \frac{S^{0}}{\left|S^{0}\right|}=FrT_{z_{2}}\left[FrT_{z_{1}}\left(e^{i\cdot \phi_{1}^{0}}\right)\cdot e^{i\cdot \phi_{2}^{1}}\right],$$

where f is a target image. We can further obtain:

(14) $$e^{i\cdot \phi_{2}^{1}}=FrT_{-z_{2}}\left[f\cdot \frac{S^{0}}{\left|S^{0}\right|}\right]/FrT_{z_{1}}\left(e^{i\cdot \phi_{1}^{0}}\right),$$

where $FrT_{-z_{2}}$ denotes the inverse Fresnel transform with the distance $z_{2}$. Next, assuming that there is a phase $e^{i\cdot \phi_{1}^{1}}$, it satisfies Equation (15):

(15) $$f\cdot \frac{S^{0}}{\left|S^{0}\right|}=FrT_{z_{2}}\left[FrT_{z_{1}}\left(e^{i\cdot \phi_{1}^{1}}\right)\cdot e^{i\cdot \phi_{2}^{1}}\right].$$

Therefore, we can obtain:

(16) $$e^{i\cdot \phi_{1}^{1}}=FrT_{-z_{1}}\left(FrT_{-z_{2}}\left(f\cdot \frac{S^{0}}{\left|S^{0}\right|}\right)\cdot {\left(e^{i\cdot \phi_{2}^{1}}\right)}^{*}\right),$$

where * is complex conjugate. Substituting the obtained $e^{i\cdot \phi_{2}^{1}}$ and $e^{i\cdot \phi_{1}^{1}}$ into Equation (12), we obtain:

(17) $$S^{1}=FrT_{z_{2}}\left[FrT_{z_{1}}\left(e^{i\cdot \phi_{1}^{1}}\right)\cdot e^{i\cdot \phi_{2}^{1}}\right].$$

Then, the above operation can be repeated to obtain $e^{i\cdot \phi_{2}^{2}},e^{i\cdot \phi_{1}^{2}},S^{2}\cdots \cdots$. When the *k*-th iteration stops, we can obtain $e^{i\cdot \phi_{1}^{k}}=e^{i\cdot \phi_{1}}$ and $e^{i\cdot \phi_{2}^{k}}=e^{i\cdot \phi_{2}}$. Then, the target image can be obtained by Equation (18):

(18) $$f=\left|FrT_{z_{2}}\left[FrT_{z_{1}}\left(e^{i\cdot \phi_{1}}\right)\cdot e^{i\cdot \phi_{2}}\right]\right|.$$

**Figure 10:**
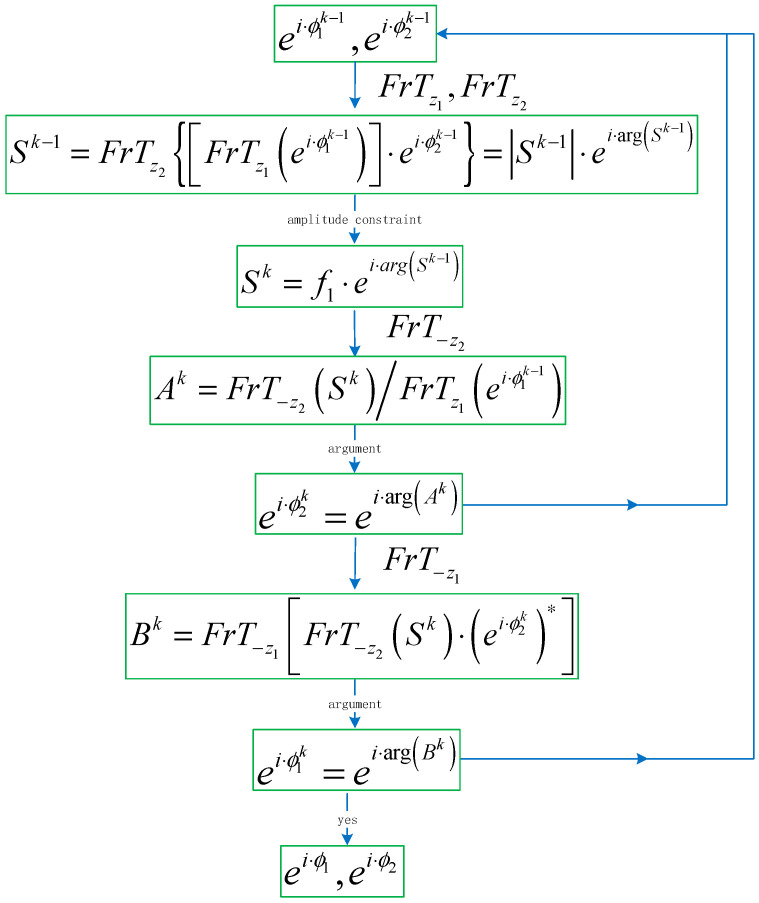
The flowchart of the double-phase retrieval (DPR) algorithm.

Through the above mathematical derivation, we give the general mathematical expression of the DPR algorithm in Equations (19)–(21):

(19) $$S^{k-1}=FrT_{z_{2}}\left[FrT_{z_{1}}\left(e^{i\cdot \phi_{1}^{k-1}}\right)\cdot e^{i\cdot \phi_{2}^{k-1}}\right],$$

(20) $$e^{i\cdot \phi_{2}^{k}}=FrT_{-z_{2}}[f\cdot \frac{S^{k-1}}{\left|S^{k-1}\right|}]/FrT_{z_{1}}\left(e^{i\cdot \phi_{1}^{k-1}}\right),$$

(21) $$e^{i\cdot \phi_{1}^{k}}=FrT_{-z_{1}}\left(FrT_{-z_{2}}\left(f\cdot \frac{S^{k-1}}{\left|S^{k-1}\right|}\right)\cdot {\left(e^{i\cdot \phi_{2}^{k}}\right)}^{*}\right),$$

k=1,2,⋯,n

where $e^{i\cdot \phi_{1}^{0}}$ and $e^{i\cdot \phi_{2}^{0}}$ are the initial random matrices. The flowchart of the DPR algorithm is shown in Figure 10.

**Figure 11:**
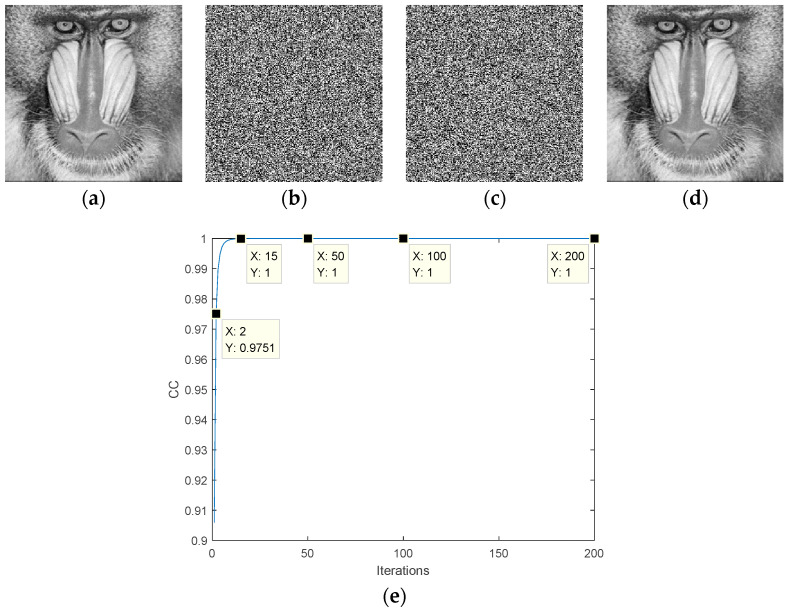
Simulation of the double-phase retrieval algorithm: (**a**) target amplitude image; (**b**) ciphertext, (**c**) ciphertext, (**d**) the recovered plaintext image, (**e**) the transformation curve of the CC.

The simulation results of the DPR algorithm are shown in Figure 11. Figure 11a is the target image. Figure 11b,c are the obtained phases, respectively. The recovered image is shown in Figure 11d. After 15 iterations, the value of CC is 1, as shown in Figure 11e. The results show that the DPR algorithm has good convergence, can obtain the exact solution, and can recover the image losslessly. We will further analyze the convergence in Section 4.

### 3.3. Multiple-Phase Retrieval Algorithm

**Figure 12:**
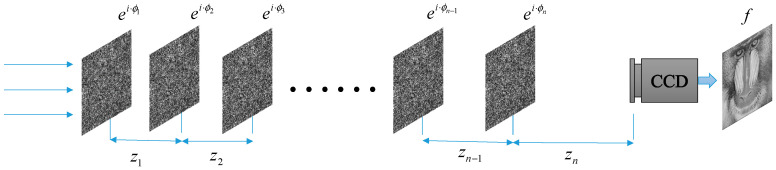
The optical system of multiple-phase modulation.

Further, we present the MPR algorithm, and its principle is shown in Figure 12. When *n* phase encodings are arranged in a certain order, the output can directly obtain the recovered image using parallel light illumination. For image encryption, an image is encrypted into *n* phase encodings.

With the help of the derivation in Section 3.2, we present the mathematical expressions for the MPR algorithm:

(22) $$S^{k-1}=FrT_{z_{n}}\left\{FrT_{z_{n-1}}\left[\cdots FrT_{z_{2}}\left(FrT_{z_{1}}\left(e^{i\cdot \phi_{1}^{k-1}}\right)\cdot e^{i\cdot \phi_{2}^{k-1}}\right)\cdots \right]\cdot e^{i\cdot \phi_{n}^{k-1}}\right\},$$

(23) $$e^{i\cdot \phi_{n}^{k}}=\frac{FrT_{-z_{n}}\left[f\cdot \frac{S^{k-1}}{\left|S^{k-1}\right|}\right]}{FrT_{z_{n-1}}\left\{FrT_{z_{n-2}}\left[\cdots FrT_{z_{2}}\left(FrT_{z_{1}}\left(e^{i\cdot \phi_{1}^{k-1}}\right)\cdot e^{i\cdot \phi_{2}^{k-1}}\right)\cdots \right]\cdot e^{i\cdot \phi_{n-1}^{k-1}}\right\}},$$

(24) $$e^{i\cdot \phi_{n-1}^{k}}=\frac{FrT_{-z_{n-1}}\left\{FrT_{-z_{n}}\left[f\cdot \frac{S^{k-1}}{\left|S^{k-1}\right|}\right]\cdot {\left(e^{i\cdot \phi_{n}^{k}}\right)}^{*}\right\}}{FrT_{z_{n-2}}\left\{FrT_{z_{n-3}}\left[\cdots FrT_{z_{2}}\left(FrT_{z_{1}}\left(e^{i\cdot \phi_{1}^{k-1}}\right)\cdot e^{i\cdot \phi_{2}^{k-1}}\right)\cdots \right]\cdot e^{i\cdot \phi_{n-2}^{k-1}}\right\}},$$

**Figure 13:**
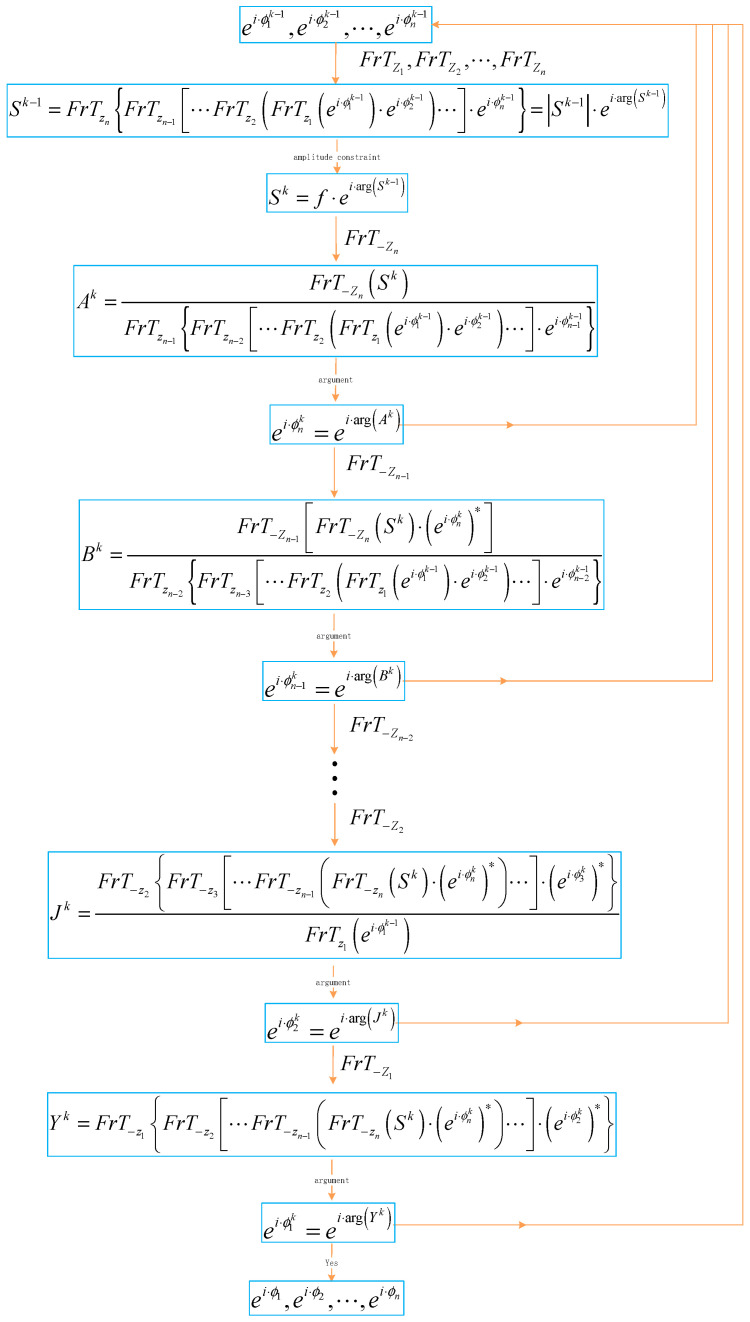
The flowchart of the multiple-phase retrieval (MPR) algorithm.

⫶

(25) $$e^{i\cdot \phi_{1}^{k}}=\arg\left(FrT_{-z_{1}}\left\{FrT_{-z_{2}}\left[\cdots FrT_{-z_{n-1}}\left(FrT_{-z_{n}}\left(f\cdot \frac{S^{k-1}}{\left|S^{k-1}\right|}\right)\cdot {\left(e^{i\cdot \phi_{n}^{k}}\right)}^{*}\right)\cdots \right]\cdot {\left(e^{i\cdot \phi_{2}^{k}}\right)}^{*}\right\}\right),$$

k=1,2,⋯,n

where $e^{i\cdot \phi_{1}^{0}},e^{i\cdot \phi_{2}^{0}},\cdots ,e^{i\cdot \phi_{n-1}^{0}}$ and $e^{i\cdot \phi_{n}^{0}}$ are the initial random phase encodings. The flowchart of the MPR algorithm is shown in Figure 13.

The SPR and DRP algorithms are special examples of the algorithm proposed in this section. The convergence of these algorithms and the comparison with the G-S algorithm is analyzed in Section 4.

## 4. Convergence

### 4.1. G-S Algorithm and Single-Phase Retrieval Algorithm

**Figure 14:**
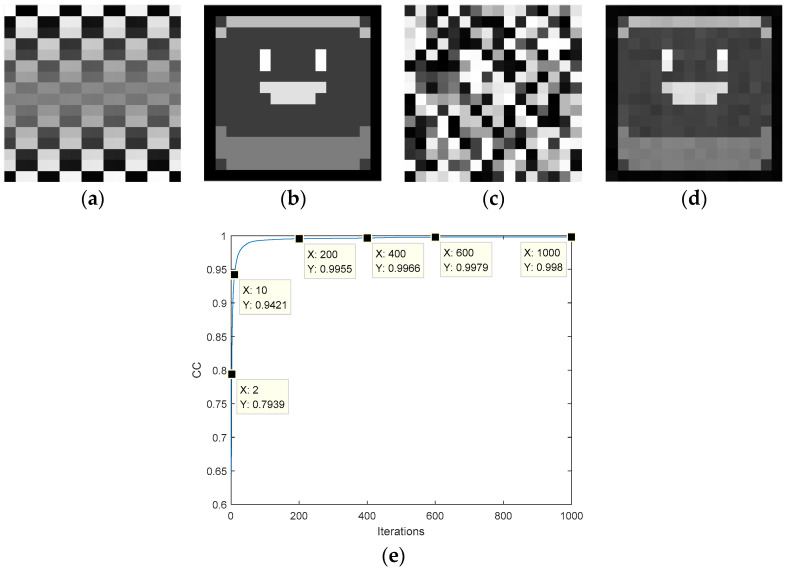
Application of the G-S algorithm: (**a**) input amplitude image, (**b**) target amplitude image, (**c**) ciphertext, (**d**) the recovered plaintext image, (**e**) the transformation curve of the CC.

The convergence of the G-S algorithm often stagnates, and the obtained solution is an approximate solution [11]. Next, we will perform an experiment by selecting two matrices *A* and *B* (both are 16 × 16). Here, matrix *A* is the magic matrix, and the corresponding imaging is shown in Figure 14a. The imaging corresponding to matrix *B* is shown in Figure 14b. We use Figure 14a as the input image and Figure 14b as the target image. The G-S algorithm performs 1000 iterations, and the obtained phase information is shown in Figure 14c. Figure 14d is the recovered image, and its corresponding pixel matrix is *C*. Figure 14e is the curve of the CC, which is closer to 1 as the number of iterations increases. However, the convergence is slow or even stagnant in the later stages of the iterative process. Our analysis shows that the G-S can recover the image well, but the exact solution is not obtained.

$$A=\left(\begin{array}{cccccccccccccccc} 256 & 2 & 3 & 253 & 252 & 6 & 7 & 249 & 248 & 10 & 11 & 245 & 244 & 14 & 15 & 241\\ 17 & 239 & 238 & 20 & 21 & 235 & 234 & 24 & 25 & 231 & 230 & 28 & 29 & 227 & 226 & 32\\ 33 & 223 & 222 & 36 & 37 & 219 & 218 & 40 & 41 & 215 & 214 & 44 & 45 & 211 & 210 & 48\\ 208 & 50 & 51 & 205 & 204 & 54 & 55 & 201 & 200 & 58 & 59 & 197 & 196 & 62 & 63 & 193\\ 192 & 66 & 67 & 189 & 188 & 70 & 71 & 185 & 184 & 74 & 75 & 181 & 180 & 78 & 79 & 177\\ 81 & 175 & 174 & 84 & 85 & 171 & 170 & 88 & 89 & 167 & 166 & 92 & 93 & 163 & 162 & 96\\ 97 & 159 & 158 & 100 & 101 & 155 & 154 & 104 & 105 & 151 & 150 & 108 & 109 & 147 & 146 & 112\\ 144 & 114 & 115 & 141 & 140 & 118 & 119 & 137 & 136 & 122 & 123 & 133 & 132 & 126 & 127 & 129\\ 128 & 130 & 131 & 125 & 124 & 134 & 135 & 121 & 120 & 138 & 139 & 117 & 116 & 142 & 143 & 113\\ 145 & 111 & 110 & 148 & 149 & 107 & 106 & 152 & 153 & 103 & 102 & 156 & 157 & 99 & 98 & 160\\ 161 & 95 & 94 & 164 & 165 & 91 & 90 & 168 & 169 & 87 & 86 & 172 & 173 & 83 & 82 & 176\\ 80 & 178 & 179 & 77 & 76 & 182 & 183 & 73 & 72 & 186 & 187 & 69 & 68 & 190 & 191 & 65\\ 64 & 194 & 195 & 61 & 60 & 198 & 199 & 57 & 56 & 202 & 203 & 53 & 52 & 206 & 207 & 49\\ 209 & 47 & 46 & 212 & 213 & 43 & 42 & 216 & 217 & 39 & 38 & 220 & 221 & 35 & 34 & 224\\ 225 & 31 & 30 & 228 & 229 & 27 & 26 & 232 & 233 & 23 & 22 & 236 & 237 & 19 & 18 & 240\\ 16 & 242 & 243 & 13 & 12 & 246 & 247 & 9 & 8 & 250 & 251 & 5 & 4 & 254 & 255 & 1 \end{array}\right)$$

$$B=\left(\begin{array}{cccccccccccccccc} 50 & 50 & 50 & 50 & 50 & 50 & 50 & 50 & 50 & 50 & 50 & 50 & 50 & 50 & 50 & 50\\ 50 & 100 & 200 & 200 & 200 & 200 & 200 & 200 & 200 & 200 & 200 & 200 & 200 & 200 & 100 & 50\\ 50 & 200 & 100 & 100 & 100 & 100 & 100 & 100 & 100 & 100 & 100 & 100 & 100 & 100 & 200 & 50\\ 50 & 100 & 100 & 100 & 100 & 100 & 100 & 100 & 100 & 100 & 100 & 100 & 100 & 100 & 100 & 50\\ 50 & 100 & 100 & 100 & 100 & 255 & 100 & 100 & 100 & 100 & 255 & 100 & 100 & 100 & 100 & 50\\ 50 & 100 & 100 & 100 & 100 & 255 & 100 & 100 & 100 & 100 & 255 & 100 & 100 & 100 & 100 & 50\\ 50 & 100 & 100 & 100 & 100 & 100 & 100 & 100 & 100 & 100 & 100 & 100 & 100 & 100 & 100 & 50\\ 50 & 100 & 100 & 100 & 100 & 230 & 230 & 230 & 230 & 230 & 230 & 100 & 100 & 100 & 100 & 50\\ 50 & 100 & 100 & 100 & 100 & 100 & 230 & 230 & 230 & 230 & 100 & 100 & 100 & 100 & 100 & 50\\ 50 & 100 & 100 & 100 & 100 & 100 & 100 & 100 & 100 & 100 & 100 & 100 & 100 & 100 & 100 & 50\\ 50 & 100 & 100 & 100 & 100 & 100 & 100 & 100 & 100 & 100 & 100 & 100 & 100 & 100 & 100 & 50\\ 50 & 150 & 100 & 100 & 100 & 100 & 100 & 100 & 100 & 100 & 100 & 100 & 100 & 100 & 150 & 50\\ 50 & 150 & 150 & 150 & 150 & 150 & 150 & 150 & 150 & 150 & 150 & 150 & 150 & 150 & 150 & 50\\ 50 & 150 & 150 & 150 & 150 & 150 & 150 & 150 & 150 & 150 & 150 & 150 & 150 & 150 & 150 & 50\\ 50 & 100 & 150 & 150 & 150 & 150 & 150 & 150 & 150 & 150 & 150 & 150 & 150 & 150 & 100 & 50\\ 50 & 50 & 50 & 50 & 50 & 50 & 50 & 50 & 50 & 50 & 50 & 50 & 50 & 50 & 50 & 50 \end{array}\right)$$

$$C=\left(\begin{array}{cccccccccccccccc} 56 & 50 & 50 & 57 & 44 & 52 & 45 & 50 & 54 & 52 & 52 & 51 & 52 & 46 & 50 & 53\\ 47 & 97 & 195 & 198 & 189 & 192 & 188 & 187 & 194 & 193 & 186 & 190 & 195 & 198 & 104 & 53\\ 51 & 186 & 97 & 98 & 101 & 96 & 102 & 108 & 98 & 97 & 103 & 100 & 100 & 98 & 185 & 58\\ 53 & 101 & 91 & 95 & 94 & 102 & 97 & 103 & 98 & 96 & 106 & 100 & 96 & 103 & 97 & 52\\ 53 & 100 & 96 & 97 & 94 & 255 & 96 & 104 & 102 & 108 & 251 & 92 & 94 & 94 & 99 & 52\\ 53 & 100 & 101 & 96 & 96 & 236 & 101 & 94 & 100 & 92 & 242 & 102 & 100 & 98 & 99 & 51\\ 50 & 100 & 101 & 94 & 97 & 101 & 98 & 95 & 101 & 95 & 92 & 101 & 96 & 99 & 99 & 51\\ 50 & 95 & 106 & 92 & 96 & 225 & 221 & 221 & 228 & 229 & 215 & 101 & 95 & 99 & 100 & 46\\ 51 & 100 & 98 & 101 & 99 & 99 & 226 & 214 & 227 & 224 & 102 & 97 & 92 & 94 & 98 & 50\\ 52 & 105 & 98 & 92 & 94 & 101 & 103 & 98 & 99 & 95 & 99 & 91 & 97 & 100 & 106 & 53\\ 51 & 93 & 96 & 101 & 94 & 96 & 103 & 98 & 98 & 99 & 102 & 97 & 96 & 95 & 107 & 54\\ 48 & 144 & 96 & 95 & 95 & 97 & 98 & 99 & 95 & 101 & 101 & 96 & 91 & 101 & 148 & 53\\ 52 & 144 & 141 & 147 & 145 & 149 & 142 & 142 & 151 & 149 & 151 & 144 & 151 & 142 & 143 & 55\\ 52 & 143 & 134 & 144 & 142 & 145 & 140 & 147 & 140 & 146 & 148 & 146 & 143 & 144 & 149 & 43\\ 53 & 89 & 145 & 142 & 146 & 147 & 145 & 143 & 144 & 148 & 148 & 143 & 147 & 140 & 98 & 44\\ 50 & 56 & 51 & 48 & 44 & 53 & 56 & 51 & 48 & 51 & 54 & 49 & 50 & 51 & 49 & 61 \end{array}\right)$$

**Figure 15:**
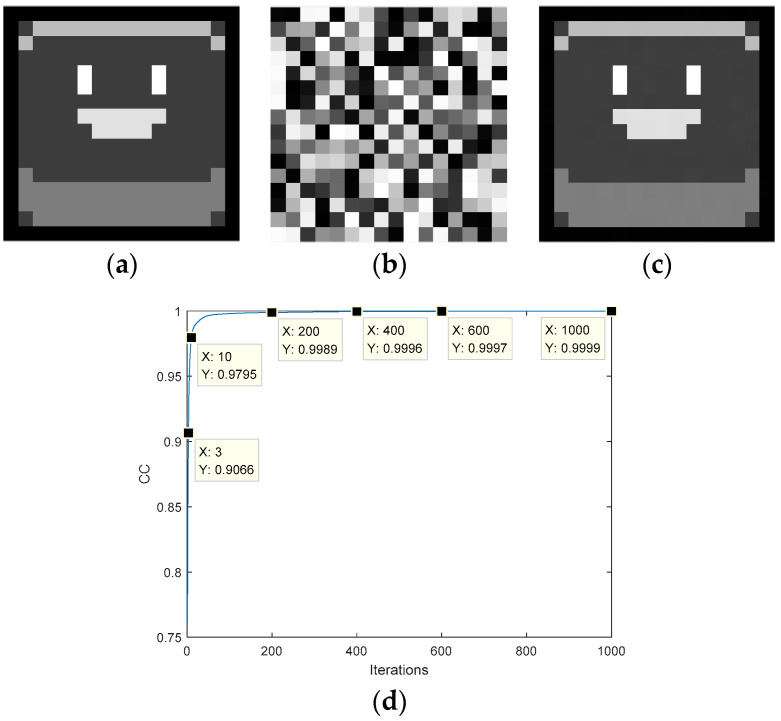
Application of the single-phase retrieval algorithm: (**a**) target amplitude image, (**b**) ciphertext, (**c**) the recovered plaintext image, (**d**) the transformation curve of the CC.

Next, we analyze the proposed SPR algorithm. Figure 15a shows that a known image is selected (matrix *B*). The phase information obtained by the SPR algorithm is shown in Figure 15b. Figure 15c is the recovered image, which is visually indistinguishable from the original image. In the restoration process, we perform 1000 iterations, and the final correlation coefficient is 0.9999, which indicates that there is no lossless recovered image. The pixel matrix of Figure 15c is *D*, which further verifies that the SPR algorithm does not obtain the exact solution.

$$D=\left(\begin{array}{cccccccccccccccc} 50 & 50 & 50 & 50 & 50 & 50 & 51 & 50 & 51 & 50 & 51 & 51 & 50 & 49 & 51 & 50\\ 50 & 100 & 199 & 200 & 199 & 200 & 199 & 200 & 200 & 200 & 199 & 299 & 200 & 199 & 100 & 50\\ 50 & 200 & 99 & 100 & 99 & 100 & 99 & 100 & 100 & 99 & 99 & 100 & 99 & 100 & 200 & 50\\ 49 & 101 & 100 & 100 & 100 & 100 & 100 & 101 & 101 & 100 & 100 & 100 & 101 & 99 & 100 & 50\\ 50 & 100 & 100 & 101 & 100 & 255 & 101 & 100 & 99 & 100 & 255 & 100 & 100 & 99 & 100 & 50\\ 51 & 101 & 100 & 100 & 100 & 255 & 100 & 101 & 100 & 100 & 254 & 99 & 100 & 100 & 100 & 51\\ 50 & 100 & 99 & 100 & 99 & 99 & 100 & 99 & 100 & 100 & 99 & 100 & 100 & 102 & 101 & 50\\ 52 & 100 & 100 & 100 & 99 & 229 & 229 & 229 & 230 & 229 & 229 & 101 & 100 & 100 & 101 & 50\\ 50 & 100 & 100 & 99 & 100 & 101 & 229 & 229 & 230 & 229 & 100 & 99 & 100 & 101 & 100 & 50\\ 50 & 99 & 101 & 100 & 100 & 99 & 101 & 101 & 101 & 100 & 100 & 100 & 101 & 100 & 99 & 51\\ 51 & 100 & 101 & 101 & 100 & 100 & 99 & 100 & 100 & 99 & 99 & 100 & 100 & 99 & 100 & 50\\ 50 & 150 & 100 & 100 & 99 & 101 & 100 & 100 & 100 & 100 & 101 & 100 & 100 & 101 & 149 & 50\\ 50 & 149 & 149 & 149 & 150 & 149 & 150 & 149 & 150 & 149 & 150 & 150 & 150 & 150 & 150 & 52\\ 50 & 150 & 150 & 149 & 150 & 150 & 151 & 151 & 151 & 150 & 150 & 149 & 150 & 149 & 150 & 51\\ 50 & 100 & 149 & 149 & 150 & 150 & 149 & 149 & 149 & 150 & 149 & 151 & 150 & 149 & 99 & 51\\ 50 & 50 & 50 & 50 & 49 & 51 & 50 & 51 & 51 & 50 & 50 & 50 & 50 & 49 & 51 & 51 \end{array}\right)$$

### 4.2. Double-Phase Retrieval Algorithm and Multiple-Phase Retrieval Algorithm

**Figure 16:**
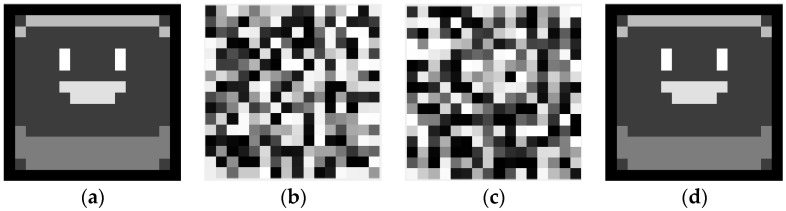

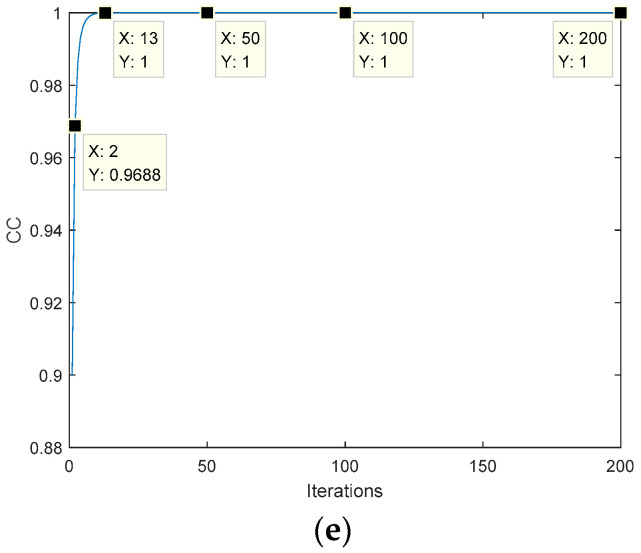
Application of the double-phase retrieval algorithm: (**a**) target amplitude image; (**b**) ciphertext, (**c**) ciphertext, (**d**) the recovered plaintext image, (**e**) the transformation curve of the CC.

The DPR algorithm is used to encrypt an image into two phase encodings. Figure 16a is a known image (matrix *B*). The obtained phase encodings are shown in Figure 16b,c. Figure 16d is the recovered image, and its pixel matrix is *E*. It can be clearly seen that the image is recovered losslessly. The CC curve is shown in Figure 16e, and the value is 1 for 13 iterations, which again verifies that the algorithm can restore information losslessly.

$$E=\left(\begin{array}{cccccccccccccccc} 50 & 50 & 50 & 50 & 50 & 50 & 50 & 50 & 50 & 50 & 50 & 50 & 50 & 50 & 50 & 50\\ 50 & 100 & 200 & 200 & 200 & 200 & 200 & 200 & 200 & 200 & 200 & 200 & 200 & 200 & 100 & 50\\ 50 & 200 & 100 & 100 & 100 & 100 & 100 & 100 & 100 & 100 & 100 & 100 & 100 & 100 & 200 & 50\\ 50 & 100 & 100 & 100 & 100 & 100 & 100 & 100 & 100 & 100 & 100 & 100 & 100 & 100 & 100 & 50\\ 50 & 100 & 100 & 100 & 100 & 255 & 100 & 100 & 100 & 100 & 255 & 100 & 100 & 100 & 100 & 50\\ 50 & 100 & 100 & 100 & 100 & 255 & 100 & 100 & 100 & 100 & 255 & 100 & 100 & 100 & 100 & 50\\ 50 & 100 & 100 & 100 & 100 & 100 & 100 & 100 & 100 & 100 & 100 & 100 & 100 & 100 & 100 & 50\\ 50 & 100 & 100 & 100 & 100 & 230 & 230 & 230 & 230 & 230 & 230 & 100 & 100 & 100 & 100 & 50\\ 50 & 100 & 100 & 100 & 100 & 100 & 230 & 230 & 230 & 230 & 100 & 100 & 100 & 100 & 100 & 50\\ 50 & 100 & 100 & 100 & 100 & 100 & 100 & 100 & 100 & 100 & 100 & 100 & 100 & 100 & 100 & 50\\ 50 & 100 & 100 & 100 & 100 & 100 & 100 & 100 & 100 & 100 & 100 & 100 & 100 & 100 & 100 & 50\\ 50 & 150 & 100 & 100 & 100 & 100 & 100 & 100 & 100 & 100 & 100 & 100 & 100 & 100 & 150 & 50\\ 50 & 150 & 150 & 150 & 150 & 150 & 150 & 150 & 150 & 150 & 150 & 150 & 150 & 150 & 150 & 50\\ 50 & 150 & 150 & 150 & 150 & 150 & 150 & 150 & 150 & 150 & 150 & 150 & 150 & 150 & 150 & 50\\ 50 & 100 & 150 & 150 & 150 & 150 & 150 & 150 & 150 & 150 & 150 & 150 & 150 & 150 & 100 & 50\\ 50 & 50 & 50 & 50 & 50 & 50 & 50 & 50 & 50 & 50 & 50 & 50 & 50 & 50 & 50 & 50 \end{array}\right)$$

**Figure 17:**
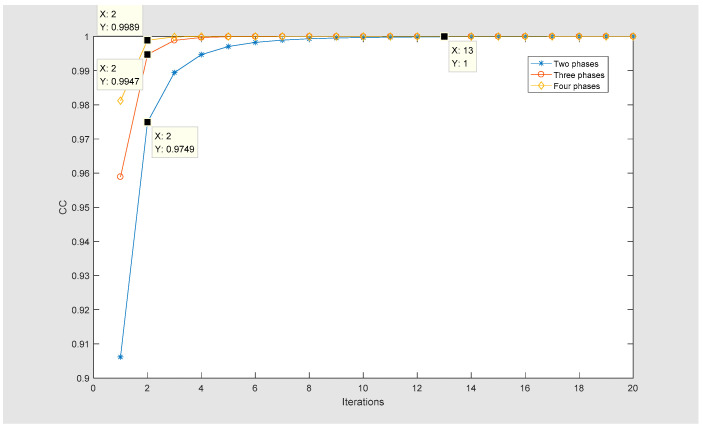
The CC curves for different numbers of phases.

The CC curve of the MPR algorithm is shown in Figure 17. The MPR algorithm can also recover information losslessly (the CC is 1), and its convergence is better than that of the DPR algorithm. Moreover, as the phase increases, the convergence speed is faster.

The G-S algorithm and the SPR algorithm are error reduction algorithms. One simply transforms back and forth between the two domains, satisfying the constraints in one before returning to the other. During the iterations, nonlinear operations are used to enforce the constraints on the amplitude to obtain the phase. It is difficult to obtain the exact solution for this kind of nonlinear operation, and the Fourier transform from one function to another is a many-to-one mapping. The DPR algorithm and the MPR algorithm proposed are linear operations in the iterative process, and the exact solution can be obtained. We take the DPR algorithm as example since it is the simplest form of MPR algorithm.

In the DPR algorithm, we finally determine the phases $e^{i\cdot \phi_{1}}$ and $e^{i\cdot \phi_{2}}$. The *k*-th iteration stops to obtain the phases $e^{i\cdot \phi_{1}}=e^{i\cdot \phi_{1}^{k}}$ and $e^{i\cdot \phi_{2}}=e^{i\cdot \phi_{2}^{k}}$, which satisfy Equation (18). In the iteration, the phase $e^{i\cdot \phi_{2}^{k}}$ is determined by Equations (14) and (15). In Equation (14), only the phase $e^{i\cdot \phi_{2}^{k}}$ is unknown, and all other terms are known. Thus, we can obtain the phase $e^{i\cdot \phi_{2}^{k}}$ in Equation (15) via a linear operation. The phase $e^{i\cdot \phi_{1}^{k}}$ can also be obtained via the linear operation of Equations (16) and (17). In this way, we finally obtained the exact solution by means of this linear operation. The MPR algorithm is an extension of the DPR algorithm, which can also obtain exact solutions. However, such a solution is not unique, and is related to the selection of the initial random phase encoding. When the initial phase encodings selected each time are different, the determined solutions are also different. This property can improve image encryption security.

## 5. Performance Analysis

### 5.1. Security and Reliability

**Figure 18:**
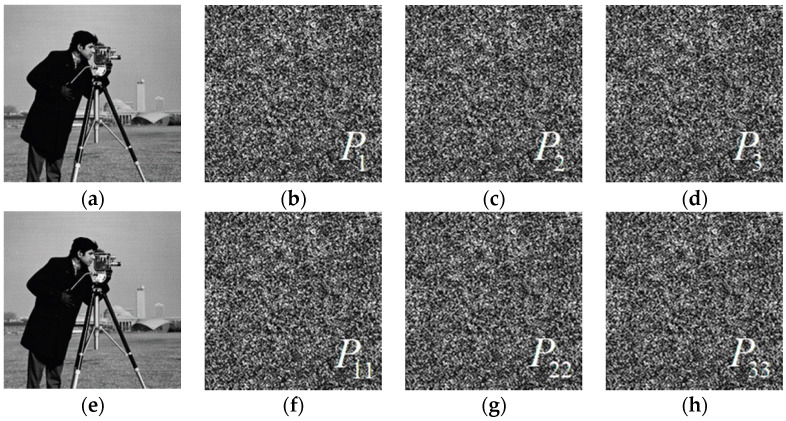
Numerical simulation of two encryptions: (**a**) plaintext; (**b**) ciphertext, (**c**) ciphertext, (**d**) ciphertext, (**e**) plaintext; (**f**) ciphertext, (**g**) ciphertext, (**h**) ciphertext.

It is well-known that the existing attack schemes can be divided into chosen ciphertext attacks, chosen plaintext attacks, known plaintext attacks, and ciphertext only attacks. In Section 4, we noted that the solution of the proposed algorithm is not unique, and this property can effectively improve the security of the system. That is, even for the same image, the ciphertexts obtained by each encryption are different. Figure 18 shows the simulation results of two encryptions. The ciphertexts P1, P2, and P3 are obtained by the first encryption. During the second encryption, we select a new set of initial phase encodings, and obtain ciphertexts P11, P22, and P33. Our research shows that only the combination of ciphertexts (P1, P2, P3) and (P11, P22, P33) can recover the plaintext while other combinations, such as (P1, P22, P3) and (P11, P2, P33), cannot recover the plaintext information.

**Figure 19:**
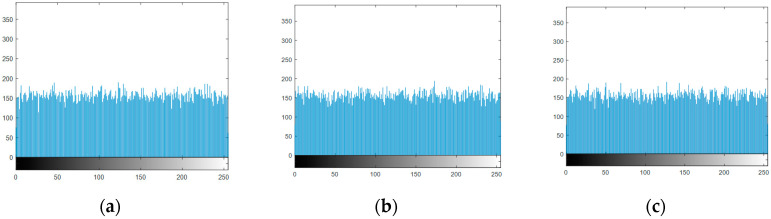
Histogram of ciphertexts: (**a**) histogram of P_1_, (**b**) histogram of P_2_, (**c**) histogram of P_3_.

A histogram is a function of the distribution of image pixels, and reflects the frequency of each pixel. The histogram of the ciphertext tends to be evenly distributed, which means that the frequency of each pixel in the ciphertext is very close, which can better cover the distribution law of the image pixels, and ensure that the algorithm can effectively resist statistical attacks. The histograms of Figure 18b–d are shown in Figure 19. The pixels of the ciphertext are evenly distributed, which means that they can resist statistical attacks.

**Figure 20:**
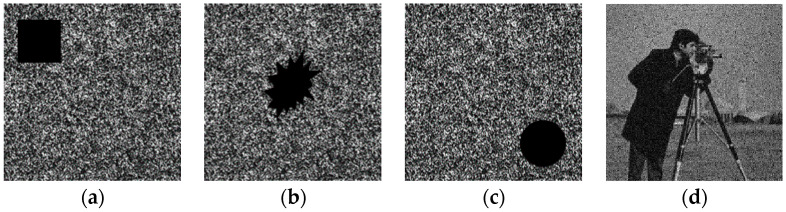
Reliability verification: (**a**) ciphertext damaged, (**b**) ciphertext damaged, (**c**) ciphertext damaged, (**d**) the recovered plaintext image.

Ciphertexts are often disturbed and lost during storage and transmission, thus, the reliability of the algorithm is particularly important. In Figure 20, we damage the ciphertexts, and then tried to recover the plaintext information from them. The recovery result is shown in Figure 20d. Even if the ciphertexts are damaged, the plaintext information can still be recovered by the proposed algorithm.

**Figure 21:**
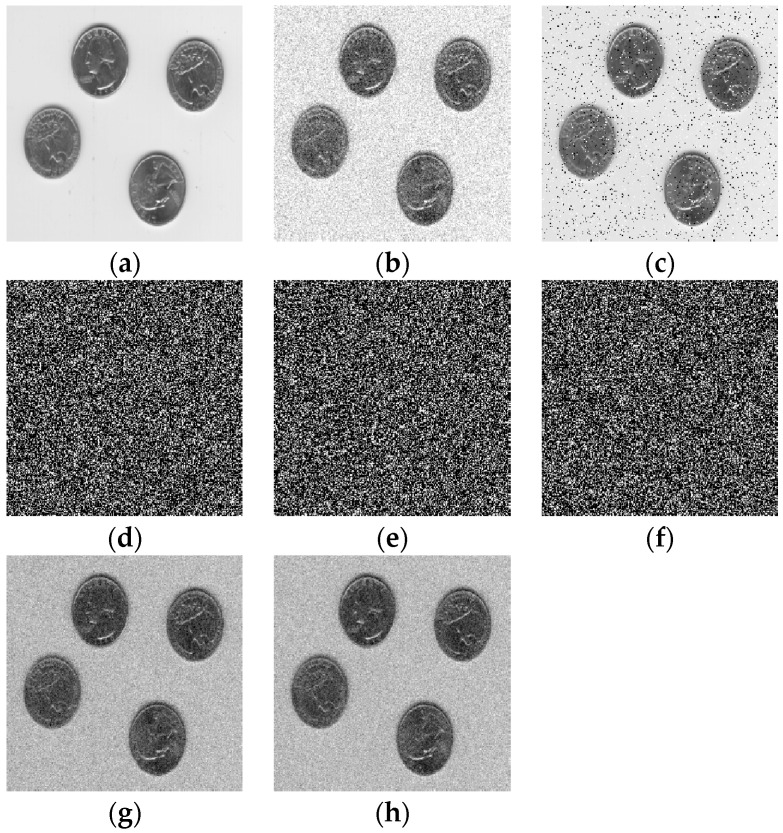
Reliability verification: (**a**) plaintext, (**b**) Gaussian noise is added, (**c**) salt and pepper noise is added, (**d**) ciphertext, (**e**) ciphertext, (**f**) ciphertext, (**g**) the recovered plaintext image, (**h**) the recovered plaintext image.

As unnecessary or redundant interference information in image data, noise seriously affects the image quality. In Figure 21, we added Gaussian noise (mean m = 0, variance var = 0.01) to the original image (Figure 21a), and the result is shown in Figure 21b. We add salt and pepper noise (noise density d = 0.05) to the original image, and the result is shown in Figure 21c. After the original image is encrypted, the ciphertexts are shown in Figure 21d–f. When the three ciphertexts are interfered by Gaussian noise, the decryption result can identify the information of the original image, as shown in Figure 21g. When the three ciphertexts are interfered by salt and pepper noise, the decryption result is shown in Figure 21h. We can still identify the original image information from the decryption result.

### 5.2. Multiple-Image Encryption

**Figure 22:**
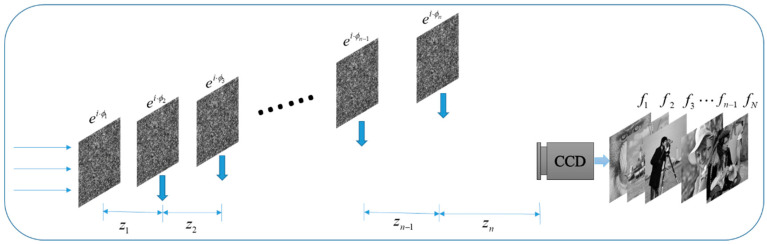
Multiple-image encryption/decryption.

With the increasing speed of information exchange, single-image encryption has gradually become unable to meet practical needs. Therefore, multiple-image encryption technology has begun to attract attention. The traditional multiple-image encryption technology generally compresses multiple plaintexts into an image or superimposes multiple plaintexts into a composite image and then encrypts them. However, these methods have difficulties achieving optical system decryption. Here, we present a novel multiple-image encryption technology using the proposed algorithm. Our idea is shown in Figure 22. When the phase $e^{i\cdot \phi_{1}}$ is placed separately in the system, the plaintext $f_{1}$ can be recovered. When phase $e^{i\cdot \phi_{1}}$ and phase $e^{i\cdot \phi_{2}}$ are placed in the system, the plaintext $f_{2}$ can be recovered. That is, as the phase increases, the recovered plaintext also increases. Finally, we can recover *n* plaintext images from *n* phase encodings.

**Figure 23:**
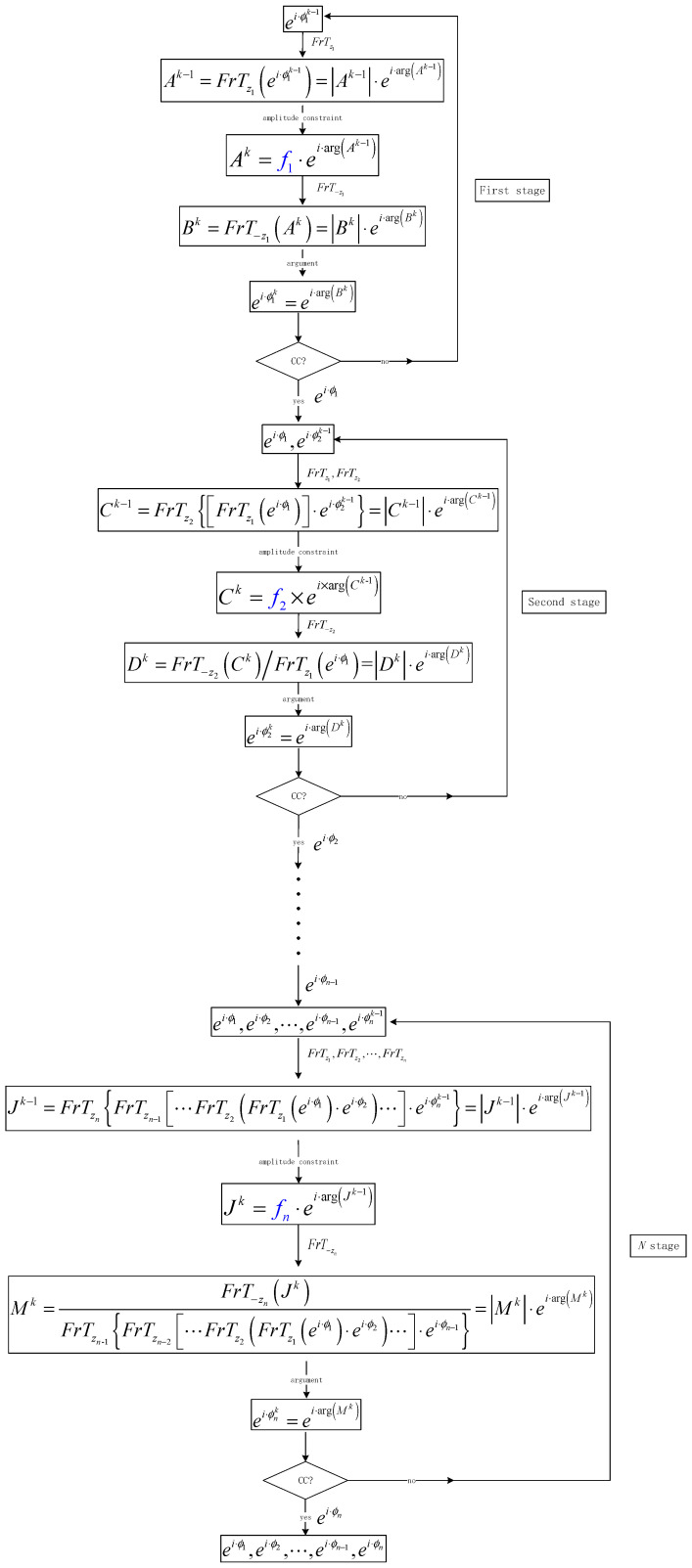
Encryption process of multiple-image encryption.

To encrypt *n* images, it is necessary to perform *N*-stage iterations, and the detailed flow chart is shown in Figure 23. In the first stage, the initial random phase $e^{i\cdot \phi_{1}^{0}}$ is input and the plaintext image $f_{1}$ is encrypted.

The CC is used to control the iteration process. When the *k*-th iteration meets certain conditions, the iteration stops and the system outputs the phase $e^{i\cdot \phi_{1}}=e^{i\cdot \phi_{1}^{k}}$(ciphertext). Then, the plaintext image $f_{1}$ can be obtained by Equation (26) as:

(26) $$f_{1}=\left|FrT_{z_{1}}\left(e^{i\cdot \phi_{1}}\right)\right|.$$

In the second stage, the plaintext image $f_{2}$ is encrypted. The phase $e^{i\cdot \phi_{1}}$ and phase $e^{i\cdot \phi_{2}^{0}}$ (random phase encoding) are used as the initial inputs. The phase $e^{i\cdot \phi_{1}}$ remains unchanged in the iteration process, and the phase $e^{i\cdot \phi_{2}}=e^{i\cdot \phi_{2}^{k}}$ is obtained when the iteration stops. The plaintext image $f_{2}$ can be obtained by Equation (27) as:

(27) $$f_{2}=\left|FrT_{z_{2}}\left(\left(FrT_{z_{1}}\left(e^{i\cdot \phi_{1}}\right)\right)\cdot e^{i\cdot \phi_{2}}\right)\right|.$$

In stage *N*, the plaintext image $f_{n}$ is encrypted. The phases $e^{i\cdot \phi_{1}},e^{i\cdot \phi_{2}},\cdots ,e^{i\cdot \phi_{n-1}}$ obtained in the previous *N*-1 stages remain unchanged as the initial phase while the initial random phase $e^{i\cdot \phi_{n}^{0}}$ is added. When the *k*-th iteration stops, the phase $e^{i\cdot \phi_{n}}=e^{i\cdot \phi_{n}^{k}}$ is obtained. Thus, the plaintext image $f_{n}$ can be obtained by Equation (28).

(28) $$f_{n}=\left|FrT_{z_{n}}\left\{FrT_{z_{n-1}}\left[\cdots FrT_{z_{2}}\left(FrT_{z_{1}}\left(e^{i\cdot \phi_{1}}\right)\cdot e^{i\cdot \phi_{2}}\right)\cdots \right]\cdot e^{i\cdot \phi_{n}}\right\}\right|.$$

**Figure 24:**
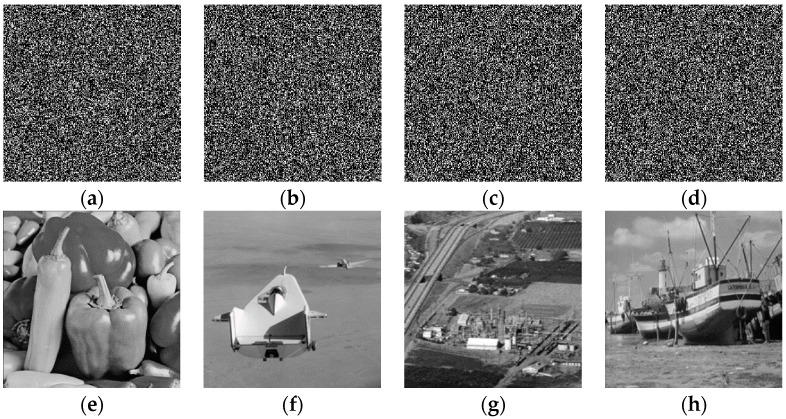
Numerical simulation of multiple-image encryption/decryption: (**a**) ciphertext, (**b**) ciphertext, (**c**) ciphertext, (**d**) ciphertext, (**e**) the recovered plaintext image by (**a**,**f**) the recovered plaintext image by (**a**,**b**,**g**) the recovered plaintext image by (**a**–**c**,**h**) the recovered plaintext image by (**a**–**d**).

The simulation verification of multiple-image encryption is shown in Figure 24. Four ciphertext images are obtained as shown in Figure 24a–d. We decrypt the ciphertext image (Figure 24a) to obtain the plaintext, as shown in Figure 24e. Figure 24f can be recovered from the ciphertexts of Figure 24a,b. Figure 24g can be recovered from the ciphertexts of Figure 24a–c. Figure 24h can be recovered from the ciphertexts of Figure 24a–d.

We have successfully recovered four plaintexts from the four ciphertexts. The numerical simulation verifies that the proposed multiple-image encryption scheme is feasible, which will allow it to provide new ideas for the application of phase retrieval algorithms.

The multiple-image encryption scheme proposed in this paper avoids the problem of crosstalk because each encryption process is carried out in succession, and each plaintext in the decryption process is obtained successively.

## 6. Discussion and Prospect

**Figure 25:**
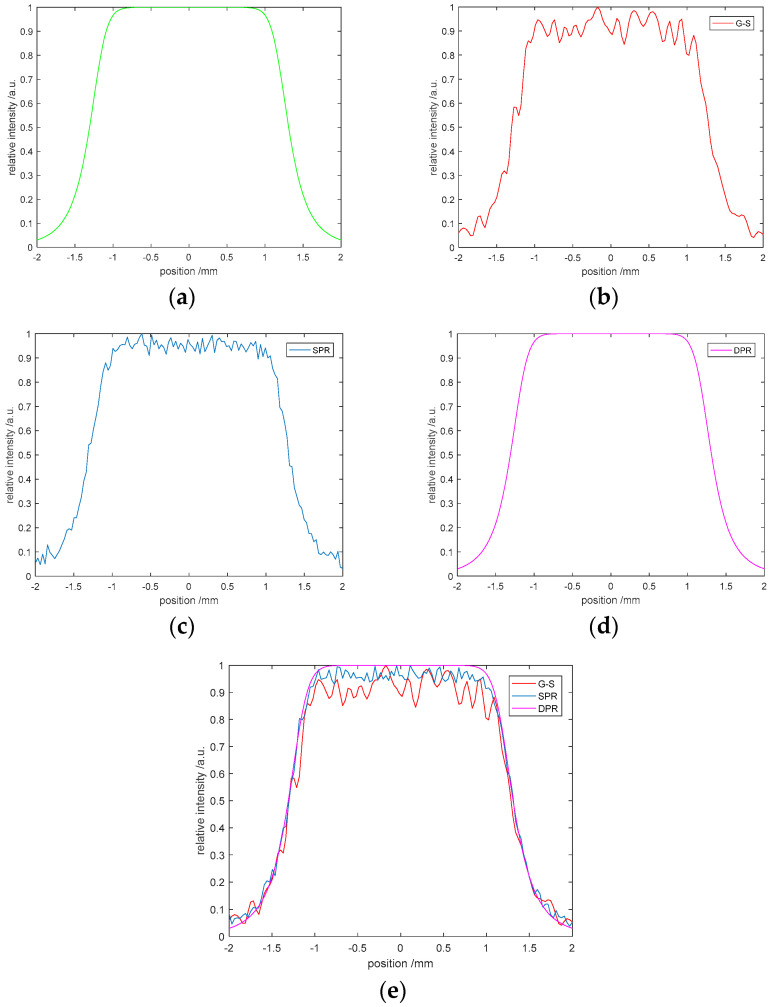
Beam shaping: (**a**) target waveform, (**b**) G-S algorithm, (**c**) SPR algorithm, (**d**) DPR algorithm, (**e**) the algorithm comparison.

The G-S algorithm is also widely used in beam shaping and binary optical element design. To improve the diffraction efficiency, many improved G-S algorithms have been proposed [42,43,44]. The main principle is to change the Gaussian beam into the desired ideal beam. Our algorithm does not need input amplitude in image encryption, so it is applied to beam shaping without a wavefront function. For our algorithm, it can be understood as turning a parallel light into an ideal beam. The waveform function we want to obtain is shown in Figure 25a. After 200 iterations with the G-S algorithm, the result is shown in Figure 25b. There is still a certain gap between this result and our objective function. The SPR algorithm is applied to 200 iterations, and the result is shown in Figure 25c, which is better than that of the G-S algorithm. Compared with the modified G-S algorithms [45,46], the SPR algorithm also improves the convergence effect. Unfortunately, these algorithms are based on a nonlinear operation, so cannot obtain accurate solutions. Our DPR and MPR algorithms are linear iterative methods based on the G-S algorithm, which can obtain accurate solutions. When the DPR algorithm is applied, the result is shown in Figure 25d, which can accurately obtain the objective function. Figure 25e shows the comparison results of the G-S, SPR, and DPR algorithms.

**Figure 26:**
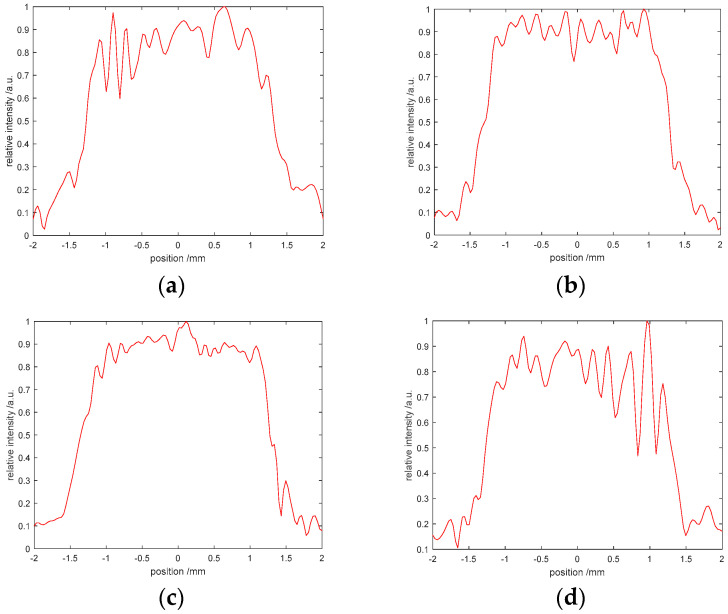
Sensitivity of initial phase based on the G-S algorithm: (**a**) the phase exp(πi/2), (**b**) the phase exp(πi), (**c**) the random phase, (**d**) the random phase.

**Figure 27:**
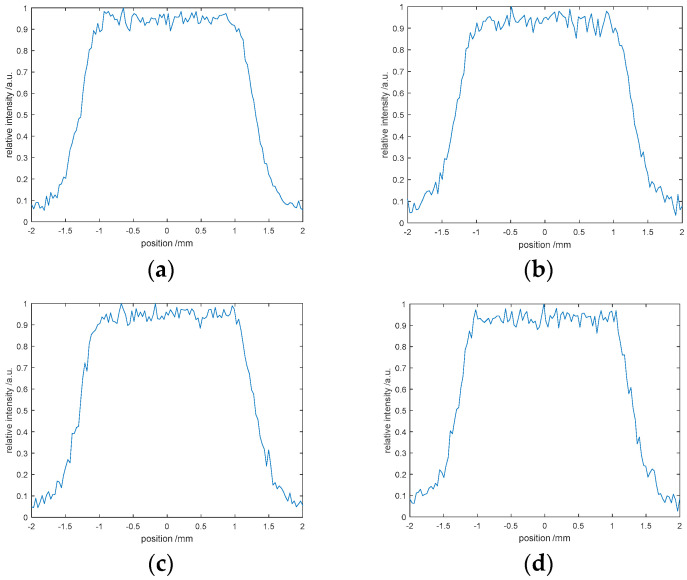
Sensitivity of initial phase based on the SPR algorithm: (**a**) the phase exp(πi/2), (**b**) the phase exp(πi), (**c**) the random phase, (**d**) the random phase.

The G-S algorithm is sensitive to the initial value, that is, the different initial phase selection has a significant impact on the results. When the initial phase is given a fixed value, the iterative results are shown in Figure 26a,b. Figure 26c,d shows the results obtained by selecting random initial phase iterations. Compared with the results in Figure 26, it can be clearly seen that the G-S algorithm is extremely sensitive to the initial phase, which directly affects the convergence effect. We use the same method to test the SPR algorithm, and the results are shown in Figure 27. It is found that the sensitivity of SPR algorithm to the initial phase is weaker.

The G-S algorithm is applied to optical image encryption because its attributes can provide good security [47,48]. The initial phase is not used as the key of the system, so different initial phases can be selected for each encryption process, and different ciphertexts can be obtained. That is, a plaintext can have many different ciphertexts, which creates difficulties in cipher decoding. Our algorithm also has such a security property.

The G-S algorithm has been used in optical cryptanalysis [49,50,51,52], but the result is not ideal because it cannot obtain the exact solution. The DPR and MPR algorithms proposed in this paper can obtain the exact solution, which clearly provides a powerful tool for optical cryptanalysis.

We have previously proposed a key distribution scheme for optical cryptography, which is also restricted by the convergence of the algorithm, and thus makes the result unsatisfactory [27]. The algorithm proposed in this paper provides an idea for key distribution.

At present, for secret image sharing, the resolution of the restored image is reduced due to sharing and the large size of the recovery image. This problem has been considered by researchers. However, the algorithms proposed in this paper can overcome this problem, which provides a new idea for secret image sharing. This is a topic that we will study next.

## 7. Conclusions

In this paper, we modify the G-S algorithm to propose the SPR, DPR, and MPR algorithms. We analyze the convergence of the proposed algorithms, and the results show that the SPR algorithm also has the same problem as the G-S algorithm. The convergence is slow in later iterations, its convergence stagnates, and only an approximate solution can be obtained. However, our analysis shows that the DPR and MPR algorithms have good convergence, can recover the plaintext without loss, and can obtain exact solutions. Furthermore, we analyzed the security and reliability of the proposed image encryption algorithm. Because different initial phase encodings can be selected for each encryption, different ciphertexts can be obtained, which improves the security of the algorithm. The reliability analysis shows that the plaintext can be recovered even if the ciphertexts are damaged. Finally, we present the multiple-image encryption scheme based on the proposed algorithm. The *n* plaintexts can be recovered from *n* ciphertexts, which greatly improves the efficiency of the system. The proposed DPR and MPR algorithms can obtain exact solutions, that is, they can recover information losslessly, which means they have an important reference value for the application of the algorithms.
